# Thrombin as Key Mediator of Seizure Development Following Traumatic Brain Injury

**DOI:** 10.3389/fphar.2019.01532

**Published:** 2020-01-14

**Authors:** Marina Ben Shimon, Efrat Shavit-Stein, Keren Altman, Chaim G. Pick, Nicola Maggio

**Affiliations:** ^1^ Department of Neurology, The Chaim Sheba Medical Center, Ramat Gan, Israel; ^2^ Department of Neurology and Neurosurgery, Sackler Faculty of Medicine and Sagol School of Neuroscience, Tel Aviv University, Tel Aviv, Israel; ^3^ Department of Anatomy and Anthropology, Sackler Faculty of Medicine and Sagol School of Neuroscience, Tel Aviv University, Tel Aviv, Israel; ^4^ Talpiot Medical Leadership Program, The Chaim Sheba Medical Center, Ramat Gan, Israel

**Keywords:** mild traumatic brain injury, epilepsy, thrombin, Protease Activated Receptor 1 (PAR1), N-Methyl-D-Aspartate (NMDA)

## Abstract

Traumatic brain injury (TBI) commonly leads to development of seizures, accounting for approximately 20% of newly diagnosed epilepsy. Despite the high clinical significance, the mechanisms underlying the development of posttraumatic seizures (PTS) remain unclear, compromising appropriate management of these patients. Accumulating evidence suggest that thrombin, the main serine protease of the coagulation cascade, is involved in PTS genesis by mediating inflammation and hyperexcitability following blood brain barrier breakdown. In order to further understand the role of thrombin in PTS, we generated a combined mild TBI (mTBI) and status epilepticus mice model, by injecting pilocarpine to mice previously submitted to head injury. Interestingly, mTBI was able to reduce seizure onset in the pilocarpine animal model as well as increase the death rate in the treated animals. In turn, pilocarpine worsened spatial orientation of mTBI treated mice. Finally, thrombin activity as well as the expression of IL1-β and TNF-α was significantly increased in the mTBI-pilocarpine treated animals. In conclusion, these observations indicate a synergism between thrombin and mTBI in lowering seizure in the pilocarpine model and possibly aggravating inflammation. We believe that these results will improve the understanding of PTS pathophysiology and contribute to the development of more targeted therapies in the future.

## Introduction

Traumatic brain injury (TBI) significantly increases the risk of seizures and is a major cause of intractable epilepsy (Hunt et al., 2013), accounting for nearly 20% of acquired epilepsy in the general population (Garga and Lowenstein, 2006). Posttraumatic seizures (PTS) may occur with a long delay of up to 20 years after the traumatic event (Piccenna et al., 2017) and involve a significant heterogeneity of seizures (Piccenna et al., 2017). The variety of PTS events includes focal seizures with or without generalization, a coexistence of both, or non-convulsive seizures (Agrawal et al., 2006). In addition to the miscellany of seizures, up to 8% of patients with PTS develop Status Epilepticus (SE) (Peets et al., 2005), a neurological emergency and significant cause of morbidity and mortality (Betjemann and Lowenstein, 2015).

Alarmingly, PTS pathophysiology is yet to be fully understood, thus management of patients and outcomes are compromised. To better understand the underlying mechanism of PTS, prior studies first tried to identify a number of risk factors involved. A major one is TBI severity (Annegers et al., 1998), especially penetrating injuries, with a significant 50% risk of epilepsy (Agrawal et al., 2006). Additional risk factors include subdural hematoma (Verellen and Cavazos, 2010) and alcohol abuse (Xu et al., 2017). However, none of them is categorical about the impact of mild TBI (mTBI) to the pathophysiology of seizures and epilepsy. As mTBI accounts for nearly 80% of the hospitalized head injury cases in the United States (Kushner, 1998), and is also related to seizure development (Webb et al., 2015), it is critical to identify the role of mTBI in the onset and progression of PTS.

To study epileptogenesis following mTBI, animal models were generated to replicate the complex implications of human mTBI and PTS. A commonly used model is the closed weight free drop mTBI rodent (Kalish and Whalen, 2016), which permits the evaluation of a great spectrum of the biological changes involved in PTS (Itsekson-Hayosh et al., 2016). Among those, blood brain barrier (BBB) breakdown (Korn et al., 2005), blood vessel disruption and vascular permeability changes (Gaetz, 2004) are crucial for PTS development. As a consequence of this circumstances, glutamate is released leading to calcium influx and therefore to development of a hyperexcitable state (Xiong et al., 2013), facilitating seizures. Additionally, intense inflammatory response, diffuse axonal injury and cell death play a central role in late structural modification and adaptive neuroplasticity occurs following TBI (Povlishock and Christman, 1995; Ziebell and Morganti-Kossmann, 2010).

The hyperexcitable state found in PTS points toward possible involvement of blood components, especially thrombin, as they were recently found to be central mediators of seizures related to BBB breakdown (Heinemann et al., 2012). Thrombin, a serine protease participating in the coagulation cascade, is originated from prothrombin cleavage by activated factor X (Krishnaswamy, 2013). After binding to its protease activated receptor (PAR1), Thrombin-PAR1 activation leads to NMDA receptors potentiation and massive calcium influx, followed by a glutamate mediated hyperexcitable state, and a lowered threshold for seizure development (Gingrich et al., 2000; Maggio et al., 2008) Critically, thrombin-PAR1 complex also participates in mTBI mechanism, causing *in vivo* synaptic disfunction and amnesia, effects that were reversed after the administration of thrombin inhibitors (Itzekson et al., 2014).

As the participation of thrombin in epileptogenesis and in BBB breakdown conditions is well known, here we chose to focus on the possibility of a synergistic effect between thrombin and mTBI in seizure development. Using a combined mice model of mTBI (free weight drop) and temporal lobe epilepsy (pilocarpine injection), we found that seizures intensity and death rate during SE were increased in the animals exposed to both treatments compared to those that experience a single one. In addition, the expression of both thrombin as well as inflammatory markers was higher in the former group than in the latter. We therefore conclude that our findings highlight the importance of thrombin in PTS pathophysiology, suggesting new directions in the management and treatment of these patients.

## Materials and Methods

### Experimental Setting

The experiments were approved by the Institutional Animal Care and Use Committee of the Sheba Medical Center which obeys to national and NIH approved rules (ANIM 1089-17). The minimal number of animals was used and all efforts were made to minimize suffering. The study was carried out in 8 weeks old male C57BL/6J mice and mTBI was induced using a free weight drop concussive device as previously described (Itzekson et al., 2014). Briefly, the device consisted of an 80-cm high metal tube (13 mm in diameter) placed vertically over the head of the mouse. Minutes prior to the injury, the animals were slightly anesthetized by isoflurane (gaseous), as previously described (Edut et al., 2014). Trauma was induced by a 50-gr metal weight dropped down the metal tube on the right anterolateral side of the head (just anterior to the right ear). The mouse was placed on a sponge immobilization board which allowed head rotation following the impact thus mimicking the natural condition of head rotation in a whiplash injury. Control mice underwent a similar procedure, however, were un-injured. Twenty-four hours after the trauma, SE was induced by a single intraperitoneal (i.p.) injection of pilocarpine hydrochloride. The trauma effect on SE development was tested in experimental settings of 250 mg/kg (low dose pilocarpine) and 350 mg/kg (high dose pilocarpine). In order to avoid side effects induced by peripheral cholinergic activation, mice were treated with atropine sulphate monohydrate (1 mg/kg, i.p.) 30 min before pilocarpine injection. Seizures were monitored and scored every 10 min using a modified Racine’s scale (0—no response, 1—freezing, 2—head nodding, 3—orofacial seizure, 4—clonic seizure, and 5—tonic seizure) (Racine, 1972), during 90 min following pilocarpine administration. After 90 min mice received diazepam (3 mg/kg; i.p.) to halt convulsions, or before in case of tonic seizure. All experimental groups received both atropine and diazepam similarly to pilocarpine treated mice.

### Open Field

Open field was performed to evaluate cognitive function and anxiety in mice upon 24 h from low pilocarpine administration. This task takes advantage of rodents aversions to large, brightly lit, open, and unknown environment. Mice were placed in the middle of the open field apparatus consisted of square box (47 cm × 47 cm × 29 cm) and given 5 min to explore. Time in the center and crossing center frequencies were recorded and analyzed using EthoVision software (Noldus information technology). The mobility index was defined as: Total duration in the center/Crossing center frequency.

### Thrombin Activity

Mice were anesthetized with pentobarbital (0.8 mg/kg) and rapidly decapitated for hippocampi dissections. Right hippocampus was dissected, and dorsal hippocampus used for thrombin activity. Thrombin enzymatic activity was measured using a fluorometric assay based on the cleavage rate of the synthetic substrate Boc-Asp (OBzl)-ProArg-AMC (I-1560; Bachem, Bubendorf, Switzerland) and defined by the linear slope of the fluorescence intensity versus time, as previously described (Bushi et al., 2013). The hippocampal tissue placed into 96-well black microplate (Nunc, Roskilde, Denmark) containing the substrate buffer. Measurements were carried out using a microplate reader (Tecan; Infinite 200; Switzerland) with excitation and emission filters of 360 nm ± 35 nm and 460 nm ± 35 nm, respectively. Reported values are normalized to protein concentration of each sample and normalized to control group values ( ±SEM).

### Gene Expression

Right hemisphere hippocampi were dissected and prepared as described before (Maggio and Segal, 2007). The RNA tissue was extracted using the TRIzol (Thermo Fisher 15596026) solubilization method followed by phase separation with chloroform. Samples were placed in 1 ml TRIzol and homogenized with bullet blender homogenizer (Next Advance) at a maximum speed for 1 min. RNA phase cleaning was performed using Bio-Rad Aurum Kit (Bio-Rad Laboratories 732-6820, Hercules, CA, USA). Total RNA (2 µg) was used for reverse transcription using high-capacity cDNA reverse transcription kit (Applied Biosystems). Quantitative real-time polymerase chain reaction was performed on the StepOne™ Real-Time PCR System (Applied Biosystems, Rhenium, Israel) using Fast SYBR Green Master (ROX) (Applied Biosystems). Hypoxanthine guanine phosphoribosyltransferase (HPRT) served as a reference gene (primers list). A standard amplification program was used (1 cycle of 95°C for 20 s and 40 cycles of 95°C for 3 s and 60°C for 30 s). The primers used in this analysis are listed in Table 1. The results were normalized to reference gene expression within the same cDNA sample and calculated using the ΔCt method with results reported as fold changes relative to control brains of sham animals and reported as mean ± SEM **Table 1**.

**Table 1:** Set of primers used for real-time PCR analysis.

Gene	Forward	Reverse
HPRT	GATTAGCGATGATGAACCAGGTT	**CCTCCCATCTCCTTCATGA CA**
Fx (factor X)	GTGGCCGGGAATGCAA	**AACCCTTCATTGTCTTCGTTAATGA**
TNFα	GACCCTCACACTCAGATCATCTTCT	**CCTCCACTTGGTGGTTTGCT**
IL1β	CTGGTGTGTGACGTTCCCATTA	**CCGACAGCACGAGGCTTT**

## Results

### mTBI Lowers Threshold for Seizures in a Pilocarpine Animal Model of SE

Administration of pilocarpine in rodents is commonly used to model SE induction and subsequent mechanisms leading to epileptogenesis (Scorza et al., 2009; Vezzani, 2009). Similarly, the weight drop model in rodents is an established mTBI model, since it mimics important characteristics of human mTBI, including PTS (Glushakov et al., 2016). In our study, we chose to combine those well-known models in order to examine the possibility that mTBI may lower seizure under epileptic prone conditions. To better examine the effect of the combined model, we used both a high dose (350 mg/kg) and a low dose (250 mg/kg) of pilocarpine.

**Figure 1:**
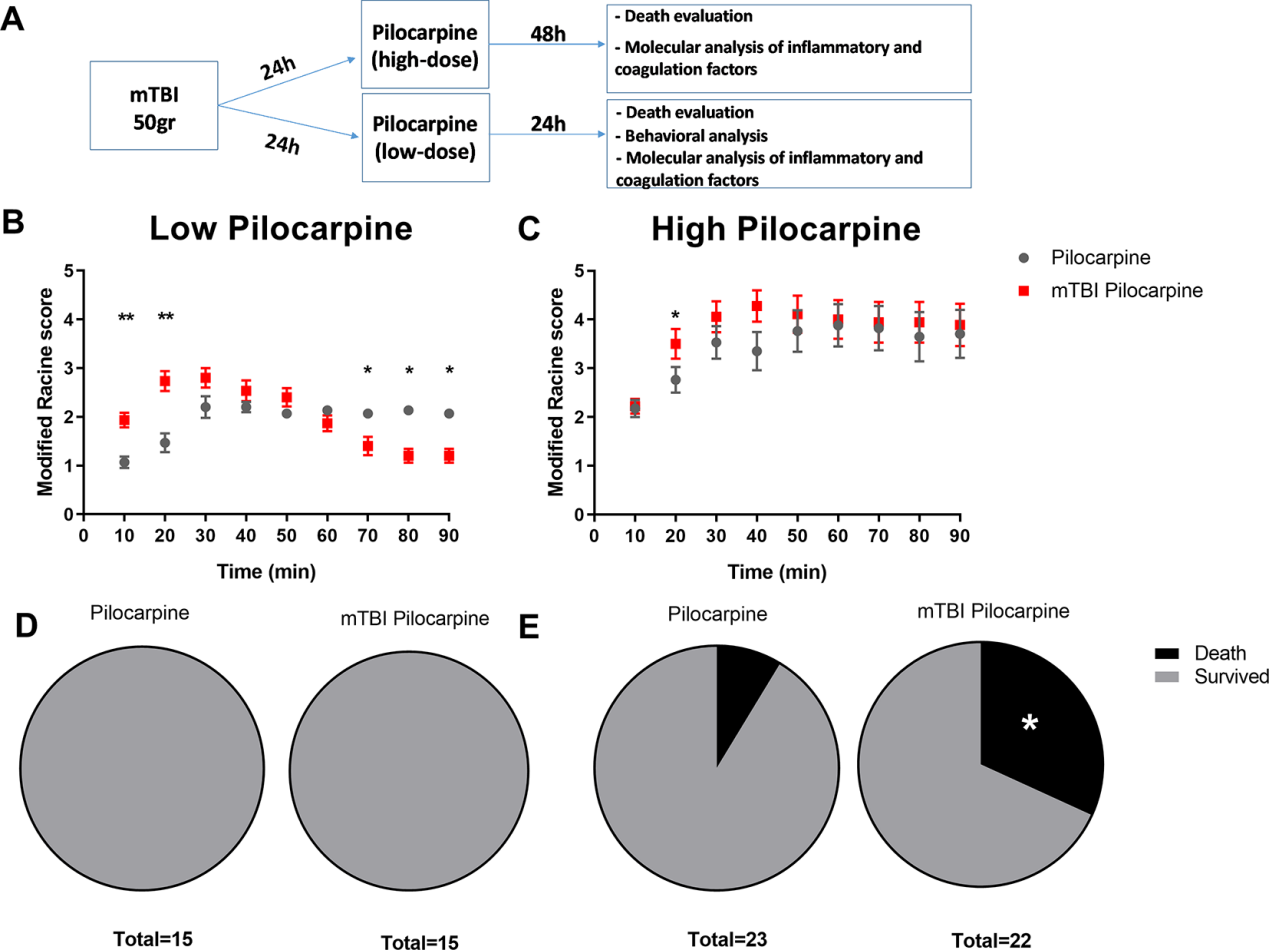
Minimal traumatic brain injury (mTBI) lowers the threshold of seizure in the pilocarpine mouse model of status epilepicus. **(A)** Timeline of the experimental procedure. **(B)** mTBI increased seizure severity upon treatment with a subthreshold low dose concentration of pilocarpine (n = 15 mice/group) while **(C)** no effect was detected when mTBI and high dose of pilocarpine were combined (n = 22-23 mice/group). While mTBI did not affect mortality in a setting of subthreshold low dose concentration of pilocarpine **(D)**, a higher mortality rate **(E)** was detected when mTBI was combined with a high dose concentration. Data is presented as mean ± SEM, ∗ p ≤ 0.05, ∗∗ p ≤ 0.01.

Primarily, we focused on the effect of both pilocarpine doses, and the consequent death that might result from those treatments. The experimental timeline included 50-gr weight drop followed by pilocarpine administration and seizure analysis (**Figure 1A**). As expected, the Racine score, used to evaluate seizure severity, was found to be worse in the high dose pilocarpine group compared to the low dose group (3.8 ± 0.45 vs. 1.9 ± 0.09, respectively, p < 0.005, at 70 min post injection, **Figures 1B, C**). Next, we chose to repeat our assessment with the mTBI and pilocarpine combined group. Interestingly, the combination of high dose pilocarpine group with mTBI did not result in a higher Racine score (**Figure 1C**). However, on the other hand, a significant difference was found when the low dose pilocarpine group was combined with mTBI (**Figure 1B**). Indeed, a low dose pilocarpine group only showed a mild seizure severity (as seen in Racine score). In contrast, the combination of low dose pilocarpine group with mTBI resulted in a higher seizure score at the onset with the highest seizures severity score achieved upon 20 min post injection (2.8 ± 0.24 vs. 1.6 ± 0.18, respectively, p < 0.004, **Figure 1B**). Strikingly, in this animal group, seizures severity dropped significantly toward the end of the experimental time window (70 min: 1.4 ± 0.22 vs. 1.9 ± 0.09 p < 0.03; 80 min: 1.2 ± 0.17 vs. 1.9 ± 0.12 p < 0.03; 90 min: 1.2 ± 0.17 vs. 1.8 ± 0.11, p < 0.05, respectively). While the groups treated with low dose pilocarpine both with and without the combination of mTBI survived the procedure (**Figure 1D**), the high dose pilocarpine combined mTBI group experienced a higher mortality rate when compared to the correspective high dose pilocarpine group (31% vs. 8.6%, **Figure 1E**). Altogether, these data show that mTBI may facilitate seizures and severity as well as worsen the epilepsy related mortality in SE.

### mTBI Worsen Cognitive Abilities in Pilocarpine Treated Animals

**Figure 2:**
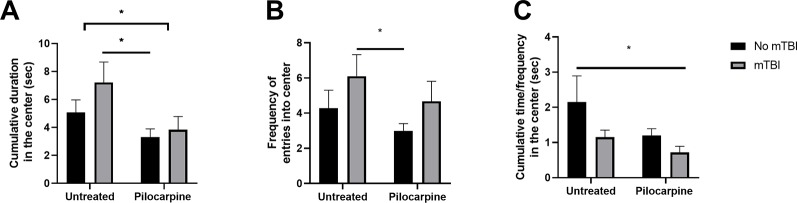
Minimal traumatic brain injury (mTBI) worsens cognitive abilities in pilocarpine treated animals. mTBI animals (n = 7–10 mice/group) exhibited a higher exploratory behavior resulting in both a higher time spent at the center of the arena **(A)** as well as in increased frequency of entries in the compartments **(B)**. In parallel, a pilocarpine treatment (n = 11 mice) resulted in a lower exploratory behavior. **(C)** A combined mTBI pilocarpine treatment (n = 6 mice) dramatically reduced the mobility index of the animals. Data is presented as mean ± SEM ∗ p ≤ 0.05.

Pilocarpine treatment as well as exposure to mTBI are both known to affect cognitive functions, i.e., spatial orientation both in humans (de Freitas Cardoso et al., 2019) and in animal models (Chauvière et al., 2009). In a previous study, the cognitive effects of an mTBI only protocol were thoroughly characterized, and showed deficits in spatial orientation (Heim et al., 2017). In this study, we studied whether a synergistic effect of both pilocarpine and mTBI might occur in worsening spatial coordination. We decided to test our hypothesis by evaluating exploratory behavior in an open field setting. Here, mTBI animals exhibited a higher exploratory behavior resulting in both a higher time spent at the center of the arena (7.2 ± 1.46 vs. 5.0 s ±0.89 s, p = 0.1, respectively, **Figure 2A**) as well as in an increased frequency of entries in this compartment (6.1 ± 1.22 vs. 4.2 ± 1.01, p = 0.15, respectively, **Figure 2B**). In parallel, treatment with pilocarpine resulted in a lower exploratory behavior compared to untreated controls. Interestingly, a combined pilocarpine and mTBI treatment resulted in a worsening of spatial orientation for mTBI treated animals (3.8 ± 0.94 vs. 7.2 s ± 1.46 s, p = 0.06; 4.6 ± 1.14 vs. 3.0 ± 0.40, p = 0.05, **Figures 2A, B**) with a dramatic reduction in their mobility index (0.7 ± 0.17 vs. 2.1 ± 0.73, respectively, p = 0.05, **Figure 2C**). These results indicate that a combined mTBI pilocarpine treatment worsens spatial orientation.

### PTS Occurrence Is Related to Thrombin Activity

**Figure 3:**
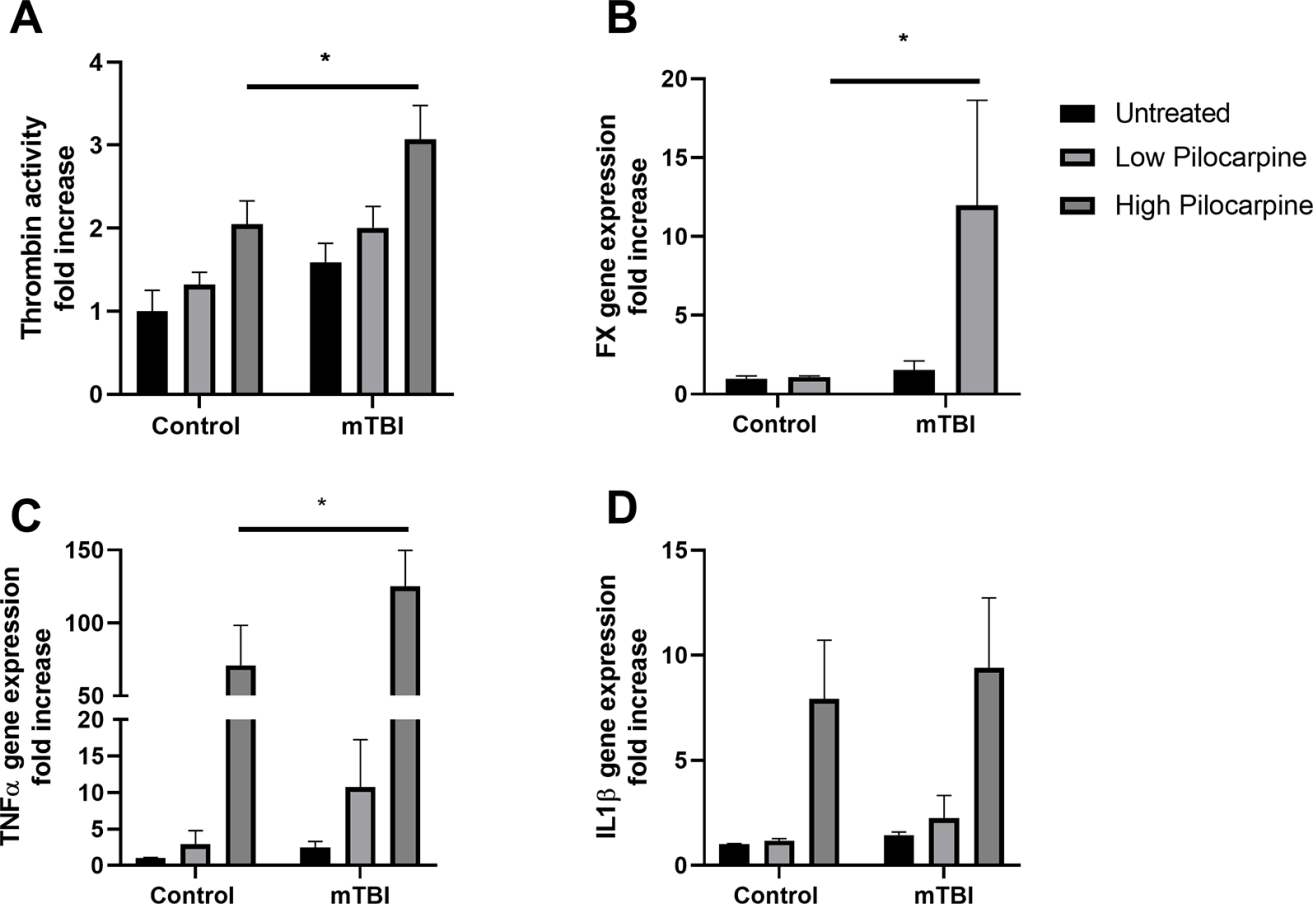
Thrombin and proinflammatory markers expression correlate with seizure severity. Thrombin activity **(A)** as well as Factor X mRNA expression **(B)** were increased in animals that underwent a combined treatment with minimal traumatic brain injury (mTBI) and pilocarpine. The mRNA expression of proinflammatory markers such as TNFa **(C)** and IL1b **(D)** were also enhanced in the same group (n = 5-19 mice/group). Data is presented as mean ± SEM, ∗ p ≤ 0.05.

To investigate the possible mechanisms involved in PTS using our mice model, we focused on thrombin which is known to play a key role both in epilepsy (Maggio et al., 2013b) and in mTBI (Xi et al., 2002). In order to investigate whether thrombin might be related to the effects of mTBI on seizures severity, thrombin activity was measured in the hippocampi of mice treated either with pilocarpine or mTBI alone, or in a combination of both. Thrombin was upregulated in all the experimental groups (**Figure 3A**) with the highest activity found in the combined groups compared to the others, (3.0 ± 0.40 and 2.0 ± 0.25 fold increase in the mTBI combined with high pilocarpine and low pilocarpine groups respectively, compared to 2.0 ± 0.27 and 1.3 ± 0.15 in the non-mTBI corresponding groups, p < 0.05) either mTBI or pilocarpine alone. Interestingly, the expression of Factor X, an enzyme responsible for the rate limiting reaction for thrombin activity, was significantly enhanced the combined group (11.98 ± 6.66 p = 0.03, **Figure 3B**). These data, might suggest a synergistic effect of mTBI and epileptic activity in upregulating thrombin levels.

### Seizures Severity Correlates With the Expression of Inflammatory Markers

Thrombin upregulation in the combined group can lead to harmful downstream effect, as thrombin has been pointed out as an important mediator of neuroinflammation and vascular disruption (Popović et al., 2012). To examine the possible effects of the thrombin upregulation, we measured the levels of proinflammatory mediators, TNF- alpha and IL1- beta that were shown to enhance brain tissue damage following trauma (Xiong et al., 2018). Interestingly, high dose of pilocarpine either alone or combined with mTBI resulted in an increased expression of both TNF-alpha (70.9 ± 27.49 and 125.1 ± 24.60 fold increase, respectively, p < 0.005) as well as IL1-beta (7.9 ± 2.80 and 9.4 ± 3.32 fold increase, respectively, p < 0.007, **Figures 3C, D**), suggesting an intense inflammatory response occurring in this setting. Contrarily, the exposure to a lower dose of pilocarpine resulted in a higher expression of inflammatory markers only in combination with mTBI (TNF-alpha: 2.8 ± 1.87 and 10.7 ± 6.50, p = 0.9, and p = 0.7, respectively; IL1-beta: 1.1 ± 0.10 and 2.2 ± 1.07, p = 0.9, and p = 0.7, respectively, **Figures 3C, D**). Altogether, these findings might highlight the possibility that seizures severity correlates with a higher expression of inflammatory markers in the brain.

## Discussion

**Figure 4:**
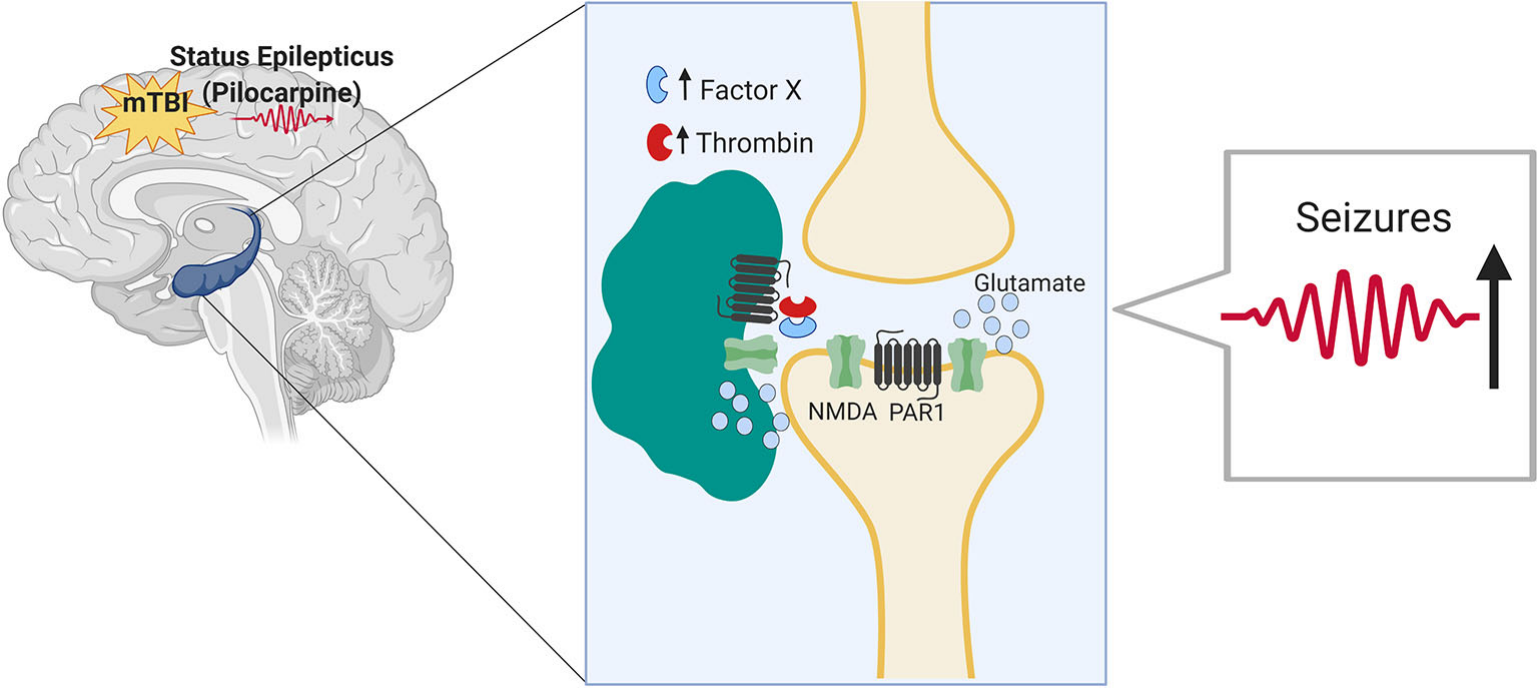
Minimal traumatic brain injury (mTBI) lowers the threshold of seizures in the pilocarpine mouse model of status epilepticus through Thrombin-PAR1 mediated excitatory pathway. Thrombin is known to rise in the brain both in hyperexcitable state as well as upon BBB breakdown following mTBI. Thrombin at high concentrations leads to the activation of NMDA receptor through a pathway mediated by PAR1. NMDA receptor activation and the subsequent massive influx of calcium lead to hyperexcitability and lower threshold for seizures to occur.

In this study, we evaluated the impact of mTBI on seizure outcomes in an animal model of seizures and epilepsy. While it is known that TBI significantly increases the risk of seizures and is a major cause of intractable epilepsy (Hunt et al., 2013), no information is currently available concerning the impact of mTBI on seizures and epilepsy. By combining two animal models, the pilocarpine model with the drop weight model of mTBI, we found that mTBI worsens seizures severity and increases epilepsy related death upon induction of SE. In addition, animals that underwent a combined treatment exhibited an impaired spatial orientation. Finally, the proteases thrombin and Factor X as well as additional inflammatory markers were upregulated in the hippocampi of the mice that underwent a combined treatment compared to the respective controls (as summarized in **Figure 4**).

Interestingly, our data show that mTBI was able to affect the seizure onset in the pilocarpine animal model of SE. While a low dose of pilocarpine is usually associated to a lower seizure’s severity score, the combination of this treatment with mTBI resulted in a faster onset, higher severity of seizures compared to controls. This finding supports the possibility that mTBI could predispose the brain such that an additional low threshold stimulus, i.e., a low dose of pilocarpine, might result in a bigger effect than if the same stimulus would have been delivered alone. In other words, mTBI could act as a “conditioning stimulus” (Statler et al., 2008; Pitkänen and Immonen, 2014) and thus affect seizures in our setting. However, if preexposure to mTBI may result in a faster seizure onset in animals treated with a low dose of pilocarpine, in the same group of animals the decay of seizure severity seems quicker. While this result may seem puzzling, a possible explanation for this phenomenon might be linked to a higher inhibitory tone in this group of animals. Indeed, as a response to the faster onset of seizures, inhibitory networks may be quickly recruited in order to shunt the over-excitatory response. Alternatively, a lower seizure onset in the low dose pilocarpine treated group might be less efficient in recruiting inhibitory networks and thus an asymptote response could arise. Additional experiments are needed to be performed in order to address this intriguing hypothesis.

Mortality due to seizures is also affected by a previous exposure to mTBI. This is an interesting observation which guarantees further exploration due to its potential clinical impact: could patients that have been exposed to mTBI and then developed epilepsy be more prone to SUDEP?

The combination of both mTBI and seizures seems to affect spatial orientation as well. Indeed, while in mTBI, spatial orientation seems to be preserved, the advent of seizures worsens it. A possible explanation of this observation may lie in the hyperexcitable state of the hippocampal network following pilocarpine treatment. As the hippocampus is the major brain area involved in spatial orientation (Burgess et al., 2002), upon seizures, the hyperexcitability of the hippocampal network might saturate the molecular and cellular mechanisms responsible for spatial orientation and memory functions (Ben Shimon et al., 2015; Maggio et al., 2017; Maggio et al., 2018) In this respect, it could be interesting to speculate about the longer time scale of this phenomenon. Our behavioral experiments were carried a day after the exposure to pilocarpine and two days after mTBI induction. Previous reports indicated that mTBI mice develop long term neuropsychiatric condition of increased anxiety and later depression (Guida et al., 2017; Belardo et al., 2019). In this sense, could a bigger time lag from the seizures restore the hippocampal abilities of the mTBI treated animals? Would the impairment in spatial orientation observed in mTBI animals upon seizure be reversible? Would a long term follow up of the impacted animals show a different behavioral phenotype, which was not observed in an mTBI only model? Future experiments are required in order to assess these hypotheses.

In several, previous contributions, we have highlighted the role of thrombin, a serine protease, in the pathogenesis of epilepsy and mTBI (Itsekson-Hayosh et al., 2016). Therefore, in this study, we aimed at assessing both thrombin activity and the expression of inflammatory markers in the mTBI-pilocarpine setting. The rationale of our assumption was that thrombin is known to rise in the brain both in hyperexcitable state as well as upon BBB breakdown following mTBI (Ben Shimon et al., 2017; De Luca et al., 2017). In the present study, thrombin was upregulated in all the experimental groups with the highest activity found in the combined mTBI-pilocarpine groups compared to the respective others. This observation was followed by the increased expression of inflammatory markers such as TNF-alpha and IL1-beta in the same group of animals, thus suggesting that this setting may cause an intense inflammatory response. Therefore, it is interesting to speculate about the possible mechanism that may link thrombin with the “conditioning” effect of mTBI on seizures. Following BBB breakdown, thrombin enters into the brain and binds to its protease activated receptor (PAR1) (Maggio et al., 2013a). Thrombin-PAR1 activation leads to NMDA receptors potentiation and massive calcium influx, followed by a glutamate mediated hyperexcitable state, and a lowered threshold for seizure development (Gingrich et al., 2000; Maggio et al., 2008). Activation of the inflammatory response may reverberate the BBB breakdown (Chodobski et al., 2011; Krenzlin et al., 2016) causing more thrombin efflux into the brain. Critically, the thrombin-PAR1 complex could also cause the impairment of spatial orientation in the treated mice due to occurrence of synaptic dysfunction and amnesia (Itzekson et al., 2014).

While additional experiments need to be performed, our observations point to an important link between mTBI and seizures. More studies are therefore needed in order to further evaluate this link for developing better therapeutical strategies for patients.

## Data Availability Statement

The datasets generated for this study are available on request to the corresponding author.

## Ethics Statement

The animal study was reviewed and approved by the Institutional Animal Care and Use Committee of the Sheba Medical Center.

## Author Contributions

ES-S supervised the project, conceived and planned the experiments, contributed to the analysis and interpretation of the results, and wrote the manuscript. MS conducted the experiments and data analysis and contributed to the writing of the manuscript. NM supervised the project, conceived and planned the experiments, gave a detailed interpretation of the results, and wrote the manuscript. KA wrote the manuscript. CP consulted regarding the project setting and results interpretation and reviewed the manuscript.

## Conflict of Interest

The authors declare that the research was conducted in the absence of any commercial or financial relationships that could be construed as a potential conflict of interest.

The handling editor declared a shared affiliation, though no other collaboration, with one of the authors, CP, at time of review.
